# Instruments for augmentative and alternative communication for children with autism spectrum disorder: a systematic review

**DOI:** 10.6061/clinics/2017/e497

**Published:** 2018-11-16

**Authors:** Jennifer Yohanna Ferreira de Lima Antão, Acary Souza Bulle Oliveira, Renata Thaís de Almeida Barbosa, Tânia Brusque Crocetta, Regiani Guarnieri, Claudia Arab, Thaís Massetti, Thaiany Pedrozo Campos Antunes, Alan Patrício da Silva, Ítalla Maria Pinheiro Bezerra, Carlos Bandeira de Mello Monteiro, Luiz Carlos de Abreu

**Affiliations:** I Laboratorio de Delineamento de Estudos e Escrita Cientifica, Faculdade de Medicina do ABC (FMABC), Santo Andre, SP, BR; II Escola Paulista de Medicina, Universidade Federal de Sao Paulo (UNIFESP/EPM), Sao Paulo, SP, BR; III Programa de Pos-Graduacao em Ciencias da Reabilitacao, Universidade de Sao Paulo, Sao Paulo, SP, BR; IV Escola Superior de Ciencias da Santa de Misericordia de Vitória (EMESCAM), Vitoria, ES, BR; V Escola de Artes, Ciencias e Humanidades, Universidade de Sao Paulo (EACH/USP), Sao Paulo, SP, BR; VI Departamento de Saude Materno Infantil, Faculdade de Saude Publica, Universidade de Sao Paulo, São Paulo, SP, BR

**Keywords:** Autism, Asperger, Education, Children, Assistive Technology

## Abstract

New technologies designed to improve the communication of autistic children can also help to promote interaction processes and cognitive and social development. The aim of this study was to analyze the instruments used to improve the communication skills of children with autism spectrum disorder. We searched the PubMed and Web of Science databases using the descriptors “autism”, “Asperger”, “education”, “children” and “assistive technology” and selected articles that met the following inclusion criteria: (i) original research; (ii) written in English; (iii) based on participants with a primary diagnosis of autism spectrum disorder; and (iv) tested an instrument designed to promote communication in children with autism spectrum disorder. Our search retrieved 811 articles, of which 34 met the inclusion criteria. Data on 26 instruments were extracted, and the measurement properties of the instruments were combined with information about their outcomes and presentation. The most commonly used interventions were the Treatment and Education of Autistic and Related Communication Handicapped Children program and the Picture Exchange Communication System. The Treatment and Education of Autistic and Related Communication Handicapped Children program was shown to produce improvements in the communication skills, socialization and self-care skills of children with autism spectrum disorder. The Picture Exchange Communication System produced inconsistent results. The results of the identified studies confirm the significant importance of these instruments in improving the communicative process of autistic children.

## INTRODUCTION

In the natural course of life, children produce verbal meanings from interactions with those around them. The exchange of information is essential to development, and social interaction is the basis of typical development. Lack of communication impairs children's development and causes problems for the people who love them 1.

Autism spectrum disorder (ASD) causes difficulties in communication and education and is characterized by a set of changes and issues relating to interaction, social communication and repetitive behavior that are usually noticed in children between 12 and 14 months old 2. In children with ASD, the development of communication has several peculiarities and does not follow the same path as in typical children 1. Another important feature of ASD is the atypical pattern of gazing during social interactions and monitoring; gaze is therefore a useful intervention target or tool 3.

The increased number of cases of ASD diagnosed worldwide 2 means that it is extremely important to develop tools to educate children with ASD and develop their communication skills. Many authors have pointed out the need to develop software and hardware that, combined with specific technology, will increase the vocabulary and communication skills of people with ASD 2,4,5.

A systematic review 2 found several tools for ASD and revealed how useful these tools are in therapy. However, these are generic tools, which means that a custom tool that meets the needs of each person is still missing, and this challenge is still very present in the interventions used today. Another study 4 found that using software with a participatory design, developed with the help of special education teachers, was an alternative way to facilitate communication and improve the social skills of children with ASD.

Furthermore, Hourcade et al. 5 found that although early intervention may occur in this population, most adults did not benefit from early intervention. However, since the launch of the iPad, there has been considerable enthusiasm in the autism community about multitouch tablets and their possible use in interventions, and hundreds of applications have been launched that may help children with ASD; however, there is little empirical evidence that any of them have positive effects. In this same study, the researchers found that children spoke more sentences, had more verbal interactions and were more physically involved with the activities when using these applications, and the authors suggested that more similar approaches should be used to increase positive social interactions in children with ASD.

Assistive technology promotes the autonomy of people with physical or cognitive disabilities, contributing to functional improvement and promoting social inclusion 1. Within the concept of assistive technology, there is a category called augmentative and alternative communication (AAC). AAC aims to develop tools that, using high or low technology, help people with orality and literacy deficits during communication and education 6.

Given how important communication is to children's development and education, it is believed that the AAC instruments used with ASD children contribute to the children's cognitive and social development and can facilitate interaction processes. Systematic reviews are needed to provide an overview of current tools and practices designed to improve the communication and education of people with ASD. Thus, the objective of this review was to analyze research on instruments for AAC and education used to improve communication skills in children with ASD.

## METHOD

This systematic review was designed in accordance with the Preferred Reporting Items for Systematic Reviews and Meta-Analyses (PRISMA) statement 7. A review of the literature was conducted in January 2018 to identify assistive technology for children with ASD.

### Search Strategy

**Figure 1:**
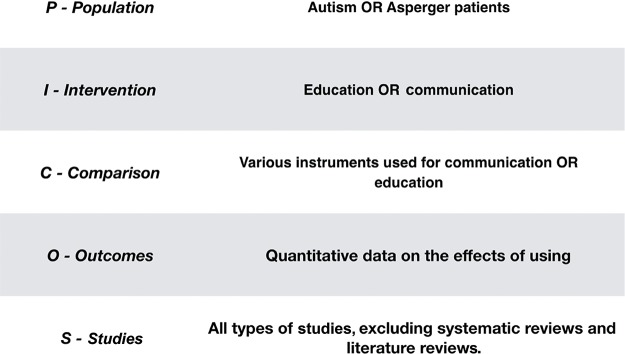
PICOS.

A comprehensive analysis of tools used to promote communication in children with ASD was conducted. This study followed the standardized PICOS (population, intervention, comparison, outcomes and studies) format (Figure 1); the search included publications on “Autism OR Asperger patients” (population) AND “Education” (intervention). The various instruments used for communication or education (comparison) were compared based on quantitative data on the effects of using the instruments with this population (outcomes).

The search for relevant articles was conducted in January 2018 and included the PubMed (http://www.ncbi.nlm.nih.gov/pubmed) and Web of Science (https://webofknowledge.com/) databases (Table 1). Two different searches were carried out in PubMed. The first search used the following search string: Autism OR Asperger AND Education AND Children. The second search used the search string Autism AND Assistive Technology AND Communication. Both searches looked for occurrences of the keywords in the title or abstract fields. The search string used on Web of Science was Autism AND Assistive Technology AND Communication; it was applied to the topic field, and the search was restricted by language (English) and type of document (article).

### Selection Process

The first step in the selection process involved reading the titles and abstracts of all the identified articles. After excluding irrelevant articles on this basis, the full text of the remaining articles was read.

### Inclusion Criteria

The literature review included original experimental articles that were written in English and based on participants with a primary diagnosis of ASD that investigated the use of a tool to promote communication in this population.

### Exclusion Criteria

Review articles, meta-analyses and editorials were excluded, and published clinical trial protocols were excluded as they do not provide data for analysis.

### Data Extraction and Quality Study

Data from the included studies were extracted using Microsoft Excel 2010. The form included the following fields, which were filled by a reviewer in the order in which they are listed below: 1 study identification (main author's name, year, and country); 2 study method (type of study, blinding, and secret allocation); 3 sample characteristics (age and gender); 4 aspects of the intervention (sample size, presence of supervision, frequency, session length, and follow-up); 5 presence of follow-up; 6 loss of follow-up; 7 reported outcomes; and 8 presented results.

## RESULTS

**Figure 2:**
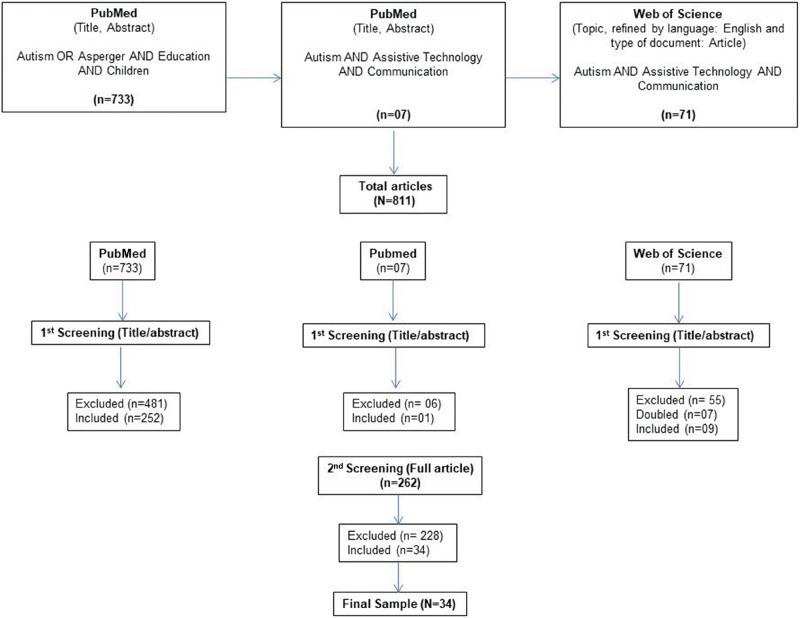
Flowchart of the Search Strategy and Selection of Articles.

The PubMed and Web of Science searches generated a total of 811 articles. After filtering by title and abstract, 262 articles were read in full, of which 34 fulfilled the inclusion criteria for this review (Figure 2). The exclusion criteria included the following: articles on the assessment of adolescents (n=9), adults (n=13), caregivers (n=13), or typical children (n=1); review/discussion articles (n=4); articles duplicated in both databases (n=7); epidemiological studies (n=59); protocol studies (n=1) (n=17); immunization studies (n=6); books (n=2); non-English articles (n=40); articles that did not intervene (n=5); articles that did not use AAC (n=247); neurology studies (n=11); studies on other diseases (n=14); information for health professionals/education presented in symposium (n=11); and informative texts (n=2).

### Study Characteristics

The 34 studies included in this review are summarized in Table 2, which provides sample data, intervention time and the instruments used to communicate with children with ASD.

The included studies were experimental, and most had samples consisting exclusively of children with autism, although some (n=5) used a control group of healthy children 8-12, and others used mixed samples including people with other health conditions, such as attention deficit hyperactivity disorder (ADHD) 10, severe mental retardation 13, invasive developmental disorder not otherwise specified (PDD-NOS) 14-18, Turner syndrome and intellectual disabilities 19, oral motor/dyspraxia, cerebral palsy, Down's syndrome, developmental delay and prematurity 20. The duration of the intervention was variable. Most of the studies used interventions consisting of multiple sessions (range: 21 to 72 sessions) at varying time intervals.

The Treatment and Education of Autistic and Related Communication Handicapped (TEACCH) program 13,14,17, and the Picture Exchange Communication System (PECS) 14-16,18,20,26, were the most used interventions. Two studies 8,28 developed and tested new tools: an interactive therapy system and the iCan application. Seok et al. 19 developed image cards that resembled images that appeared on another instrument (Korean language application) used by the authors.

### Participant Characteristics

The reviewed studies involved a total of 761 participants with ASD. The same sample was used in three studies 15,16,18, and was therefore counted only once. Other participants included healthy children 8-12 and individuals with ADHD 10, severe mental retardation 13, PDD-NOS 14-18, Turner syndrome and intellectual disabilities 19, oral motor/dyspraxia, cerebral palsy, Down's syndrome, and developmental delay, and one study included premature children 20.

### Methodological Features of the Studies

All the studies were empirical 29. The main limitations found in the selected studies were the use of an eclectic intervention and nonblinding of examiners and encoders.

### Technology

The studies predominantly used instruments for visual and auditory communication. High-tech devices, such as tablets, were used in four studies 19,28,30,31. One intervention was based on virtual reality (VR) 8. Others used the exchange of objects or the exchange of picture cards 14-16,18,20,26, that provided voice output 30, or instruments chosen by the participants 35. Several studies recorded the eye movements of children using videos 11,12,36. This information is summarized in Table 3.

## DISCUSSION

This review aimed to analyze instruments that are being used to improve the communication skills of children with ASD.

### Protocol Characteristics

Nine studies were randomized 15,16,18,23,24,26,27,31,37, and six were longitudinal 11,12,17,25,33,36. A total of 768 people with ASD participated in the studies. The limitations of the studies are discussed individually, but it is anticipated that sample size was a problem. The samples ranged from a single participant (case study) to a maximum of 198 participants, but several studies used small samples, and their results may not be reliable. Studies based on mixed samples including participants with other diseases or conditions as well as participants with ASD may produce different results, and these results cannot be generalized to the ASD population. Some studies randomly allocated participants to groups but did not blind the examiner to group status. The quality of the studies included in this review was therefore considered low, and there was little evidence of the use of validated instruments to detect changes following the interventions tested.

### Primary and Secondary Outcomes

Research has shown that the main characteristic of autism is difficulty in communicating, so it is essential to develop methods of stimulating communication and social interaction to help this population.

For the sake of convenience, our discussion was split into sections based on the techniques and devices used in the selected studies.

### 1: Techniques used for ASD

Considering the techniques found, the **TEACCH** intervention was used in eight articles, but six of those articles, in which these techniques were used individually, showed similar results and were able to prove the effectiveness of the program based on progress in the areas of communication, socialization, self-care, social reciprocity, decreased parental stress, and improved interaction between parents and children and a reduction in autistic symptoms and maladaptive behaviors 13,17,21-23,. The study by Howard et al. 14 was the only study that used the same group for TEACCH intervention plans and other image-based cards, but this group did not achieve the best results.

A meta-analysis of studies using the TEACCH intervention 38 concluded that it produces small improvements in perceptual, motor, verbal and cognitive skills but has very small or negligible effects on communication skills and daily living activities.

D'Elia et al. 17 compared TEACCH with psychomotor therapy and observed similar positive changes in language and adaptive functioning in both groups. The authors suggested that this result may have been due to the low intensity of the TEEACH intervention (four hours per week). Reviews of speech models 39 and the working management of children with ASD 40 suggested that in order to be effective, treatments must be delivered for at least 20 hours per week over at least 2 years.

Howard et al. 14 used the TEACCH program but did not obtain the best results, although the intervention was applied intensively (25-30 hours per week). In addition to the TEACCH program, the participants engaged in other activities that were incorporated into their daily routines; therefore, the children did not receive an adequate intervention due to changes in their routine.

In the study by Boyd et al. 24, the TEACCH program was compared to another technique called LEAP (Learning Experiences and Alternative Program for Preschoolers and their Parents), and there were observable gains over time, regardless of the intervention used. Regarding cognition, however, TEACCH seems to have performed better than LEAP.

Seven studies used the PECS 14-16,18,20,26,, but three of them compared it with another technique, Responsive Education and Prelinguistic Milieu Teaching (RPMT).

One comparison of the PECS and RPMT 15 found that the PECS increased nonimitative spoken communication and increased the number of different words used. Howlin et al. 26 demonstrated that the PECS improved the initiation of communication and increased the use of speech, but no effect persisted after the end of the intervention.

Lerna et al. 41 reported different results: they observed that the PECS produced improvements in joint attention, initiation and verbal requests and that these improvements were still detectable one year after the intervention, thus demonstrating that training with the PECS can promote long-term improvement.

A review of the effectiveness of the use of the PECS 42 suggested that individuals with PDD-NOS or autistic traits made better progress under the PECS than children with typical autism. The authors argued that without the quantification of autistic symptoms, the effectiveness of the PECS intervention cannot be reliably evaluated.

Complementing the results of Howard et al. 14, the group that used the PECS was unsuccessful probably because the eclectic intervention involved multiple transitions per day. Children with ASD often have difficulty adjusting to changes in their routine and with sustaining attention, so they learn better when instruction is consistent. It is also not clear that children with autism benefit from combinations of various therapies or methods for which there is limited scientific evidence of efficacy. Eldevik et al. 43 reported that children who received eclectic treatment (mixed techniques) did not perform better after one year of intervention than a group that received a behavioral intervention.

Two studies reported that when compared to the PECS, RPMT facilitated the development of communication and language skills, the exchange of objects and the initiation of joint attention 16,18. Siller and Sigman 44,45 explained the importance of joint attention for ASD, and Kasari et al. 46 emphasized that joint attention is important for social and communication skills and facilitates the development of skills such as speech. McDuffie and Yoder 47 presented an example of joint attention: during periods of development and at times when children are not communicating, parents can interact verbally with them as a language facilitation strategy. Parents can follow the example of the child in a game and describe what he or she is doing or who is playing. Sometimes, when the child does not communicate verbally, parents can use expansions as language facilitation strategies 47.

Tomas-Stonell et al. 20 used the PECS in conjunction with other devices and techniques (assistive technology devices and sign language). The authors did not specify how each of the devices and techniques was used but emphasized that there was observable improvement in communicative skills.

In the study by Fteiha 27, the PECS was compared with two other electronic devices (Language Master and the CompuThera program), and the results indicated that these devices produced higher scores for language skills than the PECS.

Another technique used with children with ASD is the ecological referential communication paradigm, which was designed to promote specific communication skills 9. This system includes the guidance of an adult as part of communicative exchange between a speaker and a receiver 48. The task was to build two identical images based on the exchange of information between the two participants, an autistic individual (speaker) and a child with typical development (receiver). The autistic individual described an image, and the child with typical development tried to build the same image based on the description 9.

Individuals with ASD have difficulty selecting and organizing information for speech and hence have problems with referential communication. Sometimes a speaker with ASD sends messages lacking information or with repetitive, ambiguous or disorganized content 49. This practice can be more effective when working with trained speakers who have the help of other support expressions, such as the specific locations of objects 50. Boada and Forns 51 also noted the importance of considering the specific location of objects, although there is no guarantee of accuracy in completing the task.

**Board games, art projects and trivia games:** Games are used to promote the socialization of all children. A study that used a social intervention that involved pairing children with ASD with developmentally typical children showed that even socially isolated ASD children could participate in activities with their partner if they were interested 35. Other studies converge with the above data and have shown that subjects with ASD increased their level of involvement and initiatives. Additionally, subjects with ASD could socialize in pairs with developmentally typical individuals since the activities were designed around their preferences 52,53. The advantage of using “natural devices” is that children with ASD can choose the task and therapists can adapt any game to make it easy and attractive to the children involved.

Other techniques (PCS and target words) are nontechnological instruments using figures, words and symbols to improve the orthographic performance of children; these instruments were used in conjunction with a speech-generating device (SGD) 34.

### 2: Devices used to help children with ASD

In addition to techniques for improving communication skills, various devices have also been used for AAC and education in children with ASD. The most commonly used devices are presented below.

**Tablet and picture cards:** Seok et al. 19 used a tablet and picture cards in their study. Chien et al. 28 also used a tablet with an application (iCAN) based on the PECS. Waddington et al. 30 used an iPad® with Proloquo2Go™, a voice application generator. One of the studies 31 used an iPad with the application Zody, a collaborative game designed to facilitate the social relationships of children. Tablets are high-technology devices that can play an important role in improving communication skills 54.

Two studies suggested that iPad® applications can be used to teach communication skills and improve language 55,56. A previous study also compared the exchange of images with high-technology device cards such as an iPad™ showed that communication acquisition varies among children with autism and on their preference for the instrument 57.

**Speech-generating devices (SGDs):** Schlosser et al. 32 and Schlosser and Blischak 34 used a LightWRITER SL35, and Schlosser et al 33 used Vantage^TM^. Both studies provided synthetic voice output and spelling feedback. Other studies in children with ASD have shown that this type of device can increase communicative interactions 58 and help adults with ASD to make requests 59.

**Nintendo Wii™:** This alternative was a game pack used in physical education classes in which the child was stimulated to play accompanied by other children, contributing to the improvement of social engagement, which was more visible in boys 37. Corroborating these findings, Hartmann and Klimmt 60 reported that girls and young women were less interested in digital games, had less knowledge about them and played less often and for shorter durations than boys.

**Microsoft Word:** In the study by Mohan et al. 61, a portable computer was used to teach communicative functions through simple copy in Microsoft Word. There was an observable increase in the quantity and quality of communication. The children came to express their needs with ease and less distraction than without the intervention. A study using computer technology to facilitate communication in children with Down's syndrome revealed that performance varied substantially. Some of the children acquired the skills needed to enter text at a productive speed, while others were very slow, and the text generated contained a substantial number of errors 62.

**Language Master:** Language Master was an electronic device used by Fteiha 27; it used cards to record a brief verbal message, which could be accompanied by audiovisual cues such as photos, words or PCS. In the same study, the **CompuThera program**, which consists of software used to develop reading skills in autistic children was evaluated. These two instruments were compared to a control group that used the PECS, and the study found that Language Master and CompuThera produced higher scores for language skills than the PECS.

**vSked:** vSked is an interactive visual scheduling system designed for elementary school classrooms that uses a custom design to understand, structure and predict daily life activities and strengthen memory and language comprehension while reducing anxiety in children with autism 63. The use of these visual artifacts in individuals with ASD contributes to reducing the symptoms associated with cognitive, communication and social deficiencies 64,65.

**Assistive technology devices and sign language:** Thomas-Stonell et al. 20 used these instruments in conjunction with the PECS. However, the authors did not specify what the instruments were or how they were used; the authors emphasized only that there was observable improvement in communication skills. A study with children with Down's syndrome who also used sign language showed positive results, corroborating the findings above 66.

**Frequency modulated (FM) system:** Schafer et al. 10 showed that an FM system has the potential to improve speech recognition and classroom behavior. Other studies demonstrated that the FM system helps speech perception and that the benefits are seen in several contexts, both outdoors and in the classroom 67-69. A study involving children with dyslexia also found that those who used the FM system showed greater improvement in reading skills and phonological awareness than a control group, which suggests that this instrument can be used in various situations 70.

**Vibrating pager:** Vibrating pagers were used in three studies 71-73 as a discrete method of warning children. Anson et al. 71 demonstrated that a “tactile prompt” (a kind of vibrating pager) improved children's behavior and engagement in independent activities in the classroom. Two other studies 72,73 found that vibrating pagers were effective in increasing verbal initiations and may promote social interaction between children with ASD and their developmentally typical peers. Tzanakaki et al. 74 corroborated the earlier findings, reporting that the use of a vibrating pager increased the number of occasions that participants initiated verbal communication with their peers.

**Looking-while-listening (LWL) and intermodal preferential looking (IPL):** The LWL paradigm was used by one study 36, while two studies 11,12 used the IPL paradigm.

Venker et al. 36 used the LWL paradigm to analyze the understanding of language of children with autism; they found that children with greater accuracy when looking at figures presented on an LCD screen processed familiar nouns faster. Considering the LWL, one study 75 found that the speed and accuracy of real-time processing of spoken language at 18 months predicted the subsequent development of vocabulary in both typically developing children and those with a limited vocabulary. Fernald et al. 76 showed that speed and accuracy in children's recognition significantly increased spoken words and were correlated with lexical and grammatical development measures.

The IPL paradigm 11, 12 involved videoing children's faces as they watched videos and using a custom program to code the children's eye movements. The children were shown two videos side by side, but they heard the soundtrack for only one of the videos. The direction and duration of a child's gaze was recorded and used as indicators of his or her understanding 77. According to results for the IPL, in the language acquisition process, the understanding of real-time issues appears to be processed the same way in children with autism and their peers with typical development, but this occurs at a later age in children with autism 11. Many autistic children are able to generalize grammatical patterns, and this capacity can be derived from previous lexical and grammatical knowledge 12.

**Interactive therapy system:** Choi et al. 8 developed an interactive therapy system that can be used in both home and hospital environments. The system is based on behavioral and cognitive therapeutic techniques and enables children to practice skills in realistic scenarios in various different contexts. The system has three components: (a) measurement of coordination skills; (b) social skills training and sensorial integration therapy; and (c) VR technology with interactive virtual scenarios. The platform was designed to allow children to interact with tangible devices in front of the screen. The therapist can control the process in real time.

VR also seemed to influence performance related to total reaction time task (the basis for many cognitive, skills and processes tasks) in children and adolescents with ASD, although these children and adolescents were slower and exhibited more anticipation than their healthy peers 78. In a study of adolescents with ASD, the use of a VR system improved social communication, performance on complex social tasks and capacity for socially appropriate conversation 79.

The tools described in this review play an important role in improving the communication skills of autistic children. However, before using the tools, an organized protocol to provide effective interventions is needed. Research has shown the importance of quantifying the severity of ASD. A method that is effective in subjects with milder symptoms, such as high-functioning ASD (Asperger syndrome), may be less effective in cases of severe ASD.

Furthermore, interventions should focus on one method at a time, without modifications to a published protocol, as children with ASD may not be able to focus on several activities at the same time. VR appears as to be a valuable tool, as children's interest is aroused by such devices, and they find them attractive and motivating.

### Limitations of the Individual Studies

The main limitation of a number of the studies was the small sample size 12,13,17,19,20,23,25,28,30. One of the limitations in the study by Schafer et al. 10 was the short period of time in which the children used the FM system, as this system is known to have greater effects when used for several hours a day.

In one study 36, the limitation was associated with different interaction devices. The authors considered that using a 58″ LCD screen was much better than using automatic screens for eye tracking, which led to data loss. Olivar-Parra et al. 9 identified the limited number of sessions and small groups as a limitation of their study. Choi et al. 8 stated that their main limitation was the impossibility of individualized therapy and the variety of content that should have been applied individually. The sample used by Panerai et al. 13 included children with severe mental retardation and autism, so their results cannot be generalized to other ASD groups.

D'Elia et al. 17 failed to compare the intervention program with other treatment models. The authors' study was not randomized, and they evaluated the participants' cognition only at baseline. Goodwin et al. 11 noted that their samples of spontaneous speech were extracted from 30-minute recordings of mother-child interactions made during six visits, and pointed out that data outside the recorded sessions were lost. Howlin et al. 26 noted that restrictions on financial and personal resources were a limitation and mentioned that the presence of the evaluators may have affected the participants' behavior.

Anson et al. 71 described a limitation of their data collection procedure, noting that it was based on a recording procedure that might underestimate the behavior of interest. Howard et al. 14 stated that their data were not collected blindly and that allocation to treatment groups was not random but determined by the children's parents. Nonrandomization of subjects 25 and the fact that the evaluators were not blinded completely was reported in some studies 24,25. Some of the studies did not explicitly report limitations 15,16,18,21,22,27,31-35,37,61,63,72,.

The tools identified in this review ranged from high-tech devices to exchange picture cards, SGDs, instruments chosen by the subjects themselves and VR systems. All the tools were effective in promoting communication and social skills, increasing the frequency of initiation of communication and joint attention, and improving the behavior of children in the classroom.

## AUTHOR CONTRIBUTIONS

Antão JY, Barbosa RT, Crocetta TB, Guarnieri R, Arab C, Massetti T Antunes TP— provided substantial contributions to the conception, design, and/or acquisition of the data, and/or the analysis and interpretation of the data. Antão JY, Oliveira AS, Barbosa RT, Crocetta TB, Guarnieri R, Arab C, Massetti T, Antunes TP Silva AP, Bezerra IM, Monteiro CB, Abreu LC were responsible for the manuscript drafting and manuscript revising for critically important intellectual content. Antão JY, Oliveira AS, Barbosa RT Crocetta TB, Guarnieri R, Arab C, Massetti T, Antunes TP, Silva AP, Bezerra IM, Monteiro CB, Abreu LC approved the manuscript final version to be published. Each author participated sufficiently in the work to take public responsibility for appropriate portions of the content. Antão JY, Oliveira AS, Barbosa RT, Crocetta TB, Guarnieri R, Arab C, Massetti T, Antunes TP, Silva AP, Bezerra IM, Monteiro CB, Abreu LC agreed to be accountable for all aspects of the work to ensure that questions related to the accuracy or integrity of any part of the work are appropriately investigated and resolved.

## Figures and Tables

**Table 1:** Search Syntax.

SEARCH SYNTAX	Number of articles
((Autism[Title/Abstract] OR Asperger[Title/Abstract]) AND Education[Title/Abstract] AND Children[Title/Abstract]	743
Topic: (Autism) AND Topic: (Assistive Technology) AND Topic: (Communication); Stipulated time: Every year; Indexes: SCI-EXPANDED, SSCI, A&HCI, CPCI-S, CPCI-SSH, ESCI	77
((Autism[Title/Abstract]) AND Assistive Technology[Title/Abstract]) AND Communication[Title/Abstract]	07

**Table 2:** Summary of selected studies that tested tools designed to promote communication in autistic children in reverse order of publication.

Reference	Sample (age in years)	Frequency and duration of intervention	Instrument	Country where the study was carried out
Fteiha, 2017	12 ASD (8-12)	One hour, 2x/week for 3 months	Language Master, CompuThera program and PECS	United Arab Emirates
Mohan et al. 2017	1 ASD (4)	45 minutes/week for 3 months	Microsoft Word	India
Dickinson; Place 2016	100 ASD (7-16)	15 minutes/day, 3x/week for 9 months	Nintendo Wii ™ and the software package ‘‘Mario & Sonic at the Olympics’’	England
Boyd et al. 2015	8 ASD (8-11)	Sessions of 15-20 minutes, 3x/week for 4 weeks	iPad (Zody)	USA
Thomas-Stonell et al., 2016	1 ASD2 oral motor/dyspraxia1 cerebral palsy2 Down’s syndrome1 developmental delay1 premature (1-5)	15 hours of intervention for 12 months	Sign language, PECS and assistive technology (NI)	Canada
Seok et al., 2015	1 ASD (8)1 Turner syndrome (16)1 intellectual disabilities (15)	52 sessions	Tablet (app not reported) and picture cards	Korea
Chien et al., 2015	11 ASD (5-16)	4 weeks	Tablet (iCan)	Taiwan
Boyd et al. 2014	198 ASD (3-5)	3 years	TEACCH and LEAP	USA
Waddington et al., 2014	3 ASD (7-10)	1x/week, 1 hour	iPad¯ (Proloquo2Go ™)	New Zealand
D'elia et al., 2014	15 ASD15 PDD-NOS (2-6)	5 hours/week (2 hours at home and 2 hours at school) for 2 years	TEACCH	Italy
Schafer et al., 2013	7 ASD (9-11)4 ADHD (10-12)11 healthy (paired by age and gender)	21 daily sessions of 45 minutes each	Personal frequency modulation system	USA
Venker et al., 2013	34 ASD (3-6)	1x/year for 3 years	Looking-while-listening	USA
Ichikawa et al., 2013	11 ASD (5-6)	Weekly 2-hour sessions, total of 20 sessions over six months	TEACCH	Japan
Koegel et al., 2012	3 ASD (9-12)	2x/week for 11 weeks	Table games; art projects; trivia games	USA
McDuffie et al., 2012*	32 ASD or PDD-NOS (1.5-5)	3x/week, 20 minutes per session, lasted for six months	RMPT vs. PECS	USA
Goodwin et al., 2012	15 ASD 18 healthy (NI age)	4-month intervals over 3 years	Intermodal preferential looking	USA
Olivar-Parra et al., 2011	20 ASD (8-23) 20 healthy (matched with respect to mental verbal age)	9 weeks	Ecological referential communication paradigm	Spain
Naigles et al., 2011	17 ASD 18 healthy (2-3.5)	2 visits at an interval of 8 months	Intermodal preferential looking	USA
Choi et al., 2010	12 ASD 20 healthy (5-6)	10 sessions	Interactive therapy system	Korea
Hirano et al., 2010	9 ASD (8-10)	3 weeks	vSked	USA
Panerai et al., 2009	34 ASD and severe mental retardation (8-9)	3 years	TEACCH	Italy
Anson et al., 2008	5 ASD (4-7)	Not reported	Vibrating pager	USA
Tsang et al., 2007	34 ASD (3-5)	12 months	TEACCH	China
Schlosser et al., 2007	5 ASD (8-10)	NI	Vantage^TM^	USA
Howlin et al., 2007	84 ASD (6-8)	5 months	PECS	UK
Yoder & Stone, 2006a*	33 ASD 3 PDD-NOS (1.5-5)	3x/week, 20 minutes for 6 months	PECS and RPMT	USA
Yoder & Stone, 2006b*	33 ASD 3 PDD-NOS (1.5-5)	3x/week, 20 minutes for 6 months	PECS and RPMT	USA
Howard et al., 2005	45 ASD 16 PDD-NOS (age not reported)	14 months	PECS and TEACCH	USA
Schlosser; Blischak, 2004	4 ASD (8-12)	NI	LightWRITER-SL35™, PCS, Target Words	USA
Panerai et al., 2002	16 ASD (>9)	NI	TEACCH	Italy
Shabani et al., 2002	3 ASD (6-7)	Not reported	Vibrating pager	USA
Schlosser et al., 1998	1 ASD (10)	21 sessions in the auditory condition, 26 in the auditory-visual condition and 31 in the visual condition	LightWRITER SL35^TM^	Canada
Taylor & Levin, 1998	1 ASD (9)	One to three 10-min sessions per day	Vibrating beeper	USA
Panerai et al., 1997	18 ASD (7-18)	1 year	TEACCH	Italy

Abbreviations: Autism spectrum disorder (ASD); Attention deficit hyperactivity disorder (ADHD); Treatment and Education of Autistic and Related Communication Handicapped Children (TEACCH); Picture Exchange Communication System (PECS); Pervasive developmental disorder not otherwise specified (PDD-NOS); Responsive Education and Prelinguistic Milieu Teaching (RPMT); Speech-generating device (SGD); Learning Experiences and Alternative Program for Preschoolers and their Parents (LEAP); Picture communication symbols (PCS). Not informed by authors (NI). Application (app). *Authors used the same sample.

**Table 3:** Objectives and characteristics of the instruments used, ordered by frequency of use.

Instrument	Apparatus	N	Objective	Characteristics
TEACCH	Tech*	08	Promote independence among people with ASD.	Intervention based on an adaptation of the physical environment, work systems, individualized schedules and use of visual aids to facilitate daily activities and promote independence.
PECS	Tech	07	Develop communicative abilities.	Participants exchange picture cards for the object depicted to distinguish figures and make sentences.
RPMT	Tech	03	Facilitate joint attention.	Toys used to encourage the child to ask for new toys and initiate joint attention.
Vibrating pager	Device	03	Emit a vibratory signal to attract the student’s attention.	Small device to provide tactile vibration, activated by a portable remote control.
Intermodal preferential looking (IPL)	Device	02	Obtain a more precise understanding of language functions in children with ASD; measure the extent to which changes in gaze are guided by the language that accompanies an image.	Two videos are displayed side by side on an LCD screen, accompanied by speech that corresponds to only one of the videos. The direction and duration of the child's eyes are recorded.
LightWRITER SL 35	Device	02	Help communication through auditory and visual stimuli.	Speech-generating device that provides synthesized voice output along with two LCD screens (listener and user), which provide orthographic feedback on the selected letters and words and a standard QWERTY keyboard.
Tablet (app not reported) and picture cards	Device	01	Practice orthography.	App: Images of animals and their names are followed by a children's song. The images are shown with the first two letters of the word corresponding to the image. Then, the whole word is shown. After this, the participant is asked to spell the word. Picture cards: 20 picture cards with pictures and animal names. Initially, two or three letters are shown, and they are different from those in the app.
Tablet (iCan)	Device	01	Develop communication, language and cognitive abilities.	PECS-based system that eliminates the process of creation and manipulation of paper picture cards, thus improving the convenience and portability of the intervention.
Board games; art projects; trivia games	Tech	01	Promote socialization among students.	Games were chosen by the participants.
Interactive Therapy System	Device	01	Practice skills, evaluate motor-visual coordination skills and stimulate sense organs.	System based on virtual reality technology with interactive virtual scenery: repetition of the desired tasks, step-by-step execution of tasks and visual and pictorial expression of social skills.
Personal frequency modulation system	Device	01	Improves the signal-to-noise ratio at the child's ear.	A small device in the children’s ears and a transmitter with a microphone to the teacher. The transmitter emits a sound to the auditory canal.
Looking-while-listening (LWL)	Device	01	Measure precision and latency of ocular movements.	Familiar images are displayed on the LCD screen on the wall and described vocally. A video camera records the children's eye movements.
Ecologic Referential Communication Paradigm	Tech	01	Analyze and practice communication abilities.	Information exchange about an image to build two identical pictures.
iPad¯ (Proloquo2Go™)	Device	01	Encourage communication functions through use of electronic voice output.	Software that generates synthetic speech output when screen icons are touched.
Microsoft Word	Device	01	Promote the improvement of communicative skills.	Laptop computer with physical keyboard that teaches different functions involving simple copy activities in Microsoft Word.
LEAP	Tech	01	Encourage the social interaction of autistic children.	Intervention that uses varied approaches to support children with autism, including learning activities and instructional strategies specifically designed to facilitate the development of functional skills, social interaction, language and adaptive behavior.
VantageTM	Device	01	Promote increases in natural speech production and improve solicitation rates for autistic children.	The Vantage™ is a speech-generating device that provides dynamic display and provides high-quality synthetic voice output and digitized voice output.
Nintendo Wii™ (Software Mario & Sonic at the Olympics)	Device	01	Improve the social functioning of children through the use of games in physical education classes.	Game pack used in physical education classes that used the features of the Wii Remote and Nunchuck motion sensor to control the actions of the on-screen character. The children were able to choose any of the game's activities that allowed up to four players simultaneously.
iPad (Zody)	Device	01	Facilitate social relationships in children with autism spectrum disorder.	Collaborative game that includes four connected mini-games. The game illustrates how good it is to work together with others and pay attention to what they are doing.
PCS	Tech	01	Assist the cognitive process (speed and accuracy) through figures and symbols.	A pictorial system consisting of designs that represent nouns, pronouns, verbs, and adjectives or that make use of symbol arrangements (e.g., clothing, shoes, goggles and gloves) used in the “grouped” and “distributed” arrangement with a visual search focus.
Target words	Tech	01	Improve children’s spelling performance.	Set of 12 words that were chosen by parents and teachers and divided into three subsets of four target words (i.e., four words for each of the three treatment conditions).
Language Master	Device	01	Improve linguistic and cognitive skills.	An electronic device that uses cards with a recordable range at the bottom. Each card is used to record a brief verbal message, which may also include matching visual cues such as photos, words, and PCS.
CompuThera	Device	01	Use educational software for language learning.	CompuThera is a computer-assisted therapy based on applied behavior analysis and discrete trial training procedures designed to teach cognitive skills to children with autism and visual learners.
vSked	Device	01	Help the individual to understand and structure their daily activities.	VSked is an interactive, collaborative visual scheduling system for autistic classrooms. VSked provides interfaces to create, facilitate, and visualize the progress of classroom activities around an interactive visual timeline.
Assistive technology devices	Device	01		The authors did not specify the instruments and did not describe how they were used.
Sign language	Tech	01	Promote language development.	Uses manual communication to transmit messages. Sign language includes simultaneous hand, finger and arm movements or body orientation and facial expressions to convey the ideas of a speaker.

Abbreviations: Number of studies (n); Autism spectrum disorder (ASD); Liquid crystal display (LCD); *Technique (Tech).
